# Dentinogenic Specificity in the Preclinical Evaluation of Vital Pulp Treatment Strategies: A Critical Review

**DOI:** 10.3390/dj3040133

**Published:** 2015-11-27

**Authors:** Dimitrios Tziafas, Konstantinos Kodonas

**Affiliations:** 1 Department of Endodontology, School of Dentistry, Aristotle University of Thessaloniki, Thessaloniki 54124, Greece; E-Mail: kkodonas@gmail.com; 2 Department of Restorative Dentistry, Endodontic Program, European University College, Ibn Sina 27D Building, DHCC Dubai, UAE

**Keywords:** histology of dental pulp, pulp capping, hard tissue bridge, reparative dentin, pulp capping materials, calcium hydroxide, Mineral Trioxide Aggregates (MTA), calcium silicate

## Abstract

Reviews on the clinical performance of vital pulp treatment strategies and capping materials repeatedly showed an insufficient grade of evidence concerning their therapeutic validity. The biological mechanisms underlying the regenerative potential of pulp-dentin complex have attracted much attention during the last two decades, since new pulp treatment modalities have been designed and tested at the preclinical level. It has been recognized that evaluation should be based on the specific ability of therapeutic interventions to signal recruitment and differentiation of odontoblast-like cells forming a matrix in a predentin-like pattern, rather than uncontrolled hard tissue deposition in a scar-like form. The aim of the present article was to critically review data from histological experimental studies on pulp capping, published during the last 7 decades. A comprehensive literature search covering the period from 1949 to 2015 was done using the Medline/Pubmed database. Inclusion of a study was dependent on having sufficient data regarding the type of capping material used and the unit of observation (human permanent tooth *in vivo* or animal permanent dentition; primary teeth were excluded). The post-operatively deposited matrix was categorized into three types: unspecified, osteotypic, or dentin-like matrix. One hundred fifty-two studies were included in the final evaluation. Data from the present systematic review have shown that only 30.2% of the 152 experimental histological pulp capping studies described the heterogenic nature of the hard tissue bridge formation, including osteotypic and tubular mineralized tissue. Structural characteristics of the new matrix and the associated formative cells were not provided by the remaining 106 studies. Analysis showed that more careful preclinical evaluation with emphasis on the evidence regarding the dentinogenic specificity of pulp therapies is required. It seems that selection of appropriate vital pulp treatment strategies and pulp capping materials would be further facilitated in terms of their therapeutic validity if international consensus could be reached on a select number of mandatory criteria for tissue-specific dentinogenic events.

## 1. Introduction

Pulpo-dentinal repair dynamics via tissue-specific mechanisms has provided operative dentistry with various treatment strategies to maintain pulp tissue in a healthy and functional state, whenever the dentin-pulp complex has been compromised by caries, trauma or restorative procedures. The techniques used for the treatment of pulp exposures in primary and permanent teeth are called direct pulp capping and pulpotomies (partial or superficial pulpotomy and full or pulp chamber pulpotomy). Direct pulp capping is indicated for small and short-term pulpal exposures of permanent teeth resulting from mechanical or traumatic injury, while partial pulpotomy is indicated for large mechanical or traumatic exposures. Full pulpotomy is the treatment of choice for long-standing exposures to the oral environment. Since both techniques share common objectives, *i.e*., to minimize reversible inflammatory reactions and to protect the pulp from the effects of further bacterial, chemical, and thermo-mechanical insults, the materials used are described as one group, pulp capping materials (PCM) in the literature, despite the fact that a few of them have only been proposed for pulpotomy situations (e.g., formocresol).

Hundreds of experimental and observational studies over the last 6 decades have focused on evaluation of various vital pulp therapy (VPT) techniques and materials [1,2]. On the other hand, numerous attempts including novel biomaterials and tissue engineering approaches with stem cells or bioactive-molecule-based applications have been designed, and their preclinical testing has been undertaken. Today it is widely accepted that the most important determinant for the long-term prognosis of vital pulp therapy is the effective control of external stimuli that can affect the underlined pulp, with the recognition that the post-operative infection is of primary importance. The present article aims to critically review the therapeutic validity of PCMs and the reliability of the criteria used in their preclinical evaluation.

### 1.1. Clinical Variables in Direct Pulp Capping and Pulpotomy

Numerous experimental and clinical studies have clearly shown that a successful outcome for vital pulp therapy is primarily dependent on the type of injury, though other variables related to the state of the dentin-pulp complex and the treatment modality have also been investigated. In general:

i. Among various clinical variables that have been accounted as factors playing a role in the outcome of the vital pulp therapy, the most important are issues related to case selection, which remains the most important parameter for the clinical success of exposed pulp treatment [1]. It is generally accepted that prognosis of direct pulp capping or pulpotomy therapies in teeth with pulp exposures remains as one of the most problematic and unpredictable methods of dental treatment. Horsted *et al.* 1985 reported that pulp survival rates of carefully selected cases treated with calcium hydroxide as capping agent was initially high (more than 80% after 5 years), but they are declining over time [3]. Pulpal exposure due to caries shows very limited potential for pulp survival due to bacterial infection of the pulp for a substantial period of time, which compromises the defense reaction [4]. In the case selection parameter, the different treatment goals of vital pulp therapy in primary and developing permanent teeth might be critically reviewed. Dental treatment of primary teeth must satisfy different goals than treatment for mature permanent teeth, due to the limited life span of primary teeth and their possible relationship to the permanent tooth successor. Although recent advances in primary tooth biology clearly demonstrated that these teeth have also a potential for wound healing with tertiary dentin formation [5], the criteria used for evaluation of PCM have not been re-evaluated and in many cases PCM with different properties are used. Similarly, dental treatment of immature permanent teeth must satisfy different goals than treatment for mature permanent teeth, due to the central role of the pulp in the physiological continuation of root development and in further deposition of primary dentin which strengthens the root dentinal walls. Thus, preservation of pulp vitality is particularly important in the immature permanent teeth, even with very different treatment indications. The absence of toxicity in PCMs and their further ability to minimize pulp inflammation and enhance pulp healing has been recognized as an important factor in the outcome of VPT [6,7,8].
ii. It has been recognized that dental pulp responds to external irritation with the set of stereotypic defensive mechanisms of the connective tissues. Whenever dentin and pulp is affected by caries, a network of inflammatory reactions of pulpal cells, micro-circulation and nerves, restorative procedures and trauma directly affects the outcome of the fundamental defensive mechanisms in the dental pulp. In patho-physiological terms the most significant difference between dental pulp and other connective tissues is the low compliance environment of the dentinal walls and the relatively constant pulp tissue volume [9]. Initial vascular reactions during pulp inflammation (vasodilatation and increased vessel permeability) taking place in the rigid enclosed pulp chamber create conditions of increased hydrostatic tissue pressure. Local reflex reactions due to activation of sensory nerve fibers and subsequent release of vasoactive peptides might be beneficial to the pulp organ under low-grade tissue irritations [10]. However, under prolonged irritation, and despite the oedema-preventing mechanisms [9], dental pulp pressure can quickly suffer irreversible damage. Thus dental pulp healing does not always follow the sequence of events taking place normally in other connective tissues. Since pulp repair is strongly dependent on a number of factors, exacerbation of an initial inflammatory reaction very often leads to general tissue necrosis.
iii. It is well-known that pulpal wound healing depends largely on the extent to which infection can be avoided [2]. Control of pre-operative infection seems to be a prerequisite for the success of vital pulp therapy. Furthermore, the control of post-operative infection depends largely on the integrity of restoration and the ability of healed dentin-pulp complex to withstand the leaking oral bacteria. Thus, the nature of the healing mechanism determines the therapeutic validity of each vital pulp treatment modality and the PCM used. The role of physico-chemical and/or biological properties of PCM in the effective control of post-operative infection still remain an unknown clinical concern.

In order to explore our understanding of therapeutic validity of the PCM and its role in the successful outcome of a given treatment modality, the knowledge on the reparative potential of the treated dental pulp, the biology of tertiary dentinogenesis and regenerative dynamics of dentin-pulp complex are briefly reviewed.

### 1.2. Dentinogenesis in Health and Disease

Only the cells of the embryonic precursor of dental pulp, the dental papilla, possess the ability to differentiate into odontoblastic cells forming primary dentin [11]. No other population of adult mesenchymal cells has the ability to differentiate into odontoblasts and this specific ability seems to be acquired by morphogenic influences during tooth development [12,13]. Odontoblasts physiologically form a dentin matrix during tooth development (primary dentin) and post-developmentally throughout of their lifespan (secondary dentin). Odontoblasts are further able to respond to exogenous stimuli, forming the reactionary type of tertiary dentin. After the destruction of primary odontoblasts, pulp cells which become new odontoblasts (odontoblast-like cells) form the reparative type of tertiary dentin. Tertiary dentin of both types, forming as a part of the wound healing mechanism in the pulp environment or in response to specific molecular signal, repairs the pulp-dentin complex in a specific spatial pattern [14,15]. This seems to be in line with the critical biological validity of a modality used in VPT. The other types of mineralized matrices in the repairing pulp environment, named fibrodentin or osteodentin, might be distinguished from tertiary dentin [16]. Apparently, these products represent defensive matrices of atypical scar-like form rather than pulp-specific tissues. However, the stereotypic processes taking place in defensive mechanisms cannot be clinically controlled and their end-result cannot be predicted [17]. Atypical hard tissue barriers are characterized by their unspecific nature and their porosity due to the osteotypic appearance of the secreted matrix. Both outcomes might be recognized as potential failures of the VPT. The atypical hard tissue has no barrier effect; it is formed at the expense of the dental pulp leading to pulp obliteration, and it is not effective in protecting the pulp from leaking bacterial threats.

### 1.3. The Reparative and Regenerative Potential of Traumatized Dentin-Pulp Complex

The irritation of the traumatized dental-pulp complex due to acute physical or mechanical trauma, including the trauma of pulp exposure, usually causes reversible pulpal damage. In the absence of chronic irritation, the dental pulp can be recovered [18]. As has been already stated, contamination of the amputated pulp with oral bacteria has been widely recognized as the most critical factor for continuing pulp inflammation and necrosis [4]. Subsequently, the ability of PCM to provide an effective barrier effect able to oppose the external irritation of the pulp-dentin complex must be directly associated with its therapeutic validity. From the first introduction of pulp capping as a treatment alternative for the exposed dental pulp, the main interest has been focused on the dentinogenic potential of pulp cells and how the PCM can release morphogenetic factors from the dentin matrix [19] and/or induce cell proliferation, migration, adhesion and differentiation, *i.e*., the formation of a dentinal barrier at the exposure site. It might be noticed here that many observational and experimental studies have documented that the exposed dental pulp can survive even in the absence of dentinal barrier [1]. Taking this into consideration, the ability of dental materials to provide the necessary barrier effect through “hermetic” sealing of the exposure site has been also investigated as the basic criterion for the therapeutic validity of a given pulp treatment modality, despite the fact that many investigators stated that in this case the risk for pulp infection, inflammation and necrosis is extremely high. Of course, it is reasonable to conclude that combination of the dentino-inductive and sealing properties in one PCM should represent the ideal solution. In any case, the inductive properties seem to be among the primary requirements from the PCM.

The nature of the dentinal barrier has been progressively recognized as a further criterion in evaluating the reliability of PCM, which needs to be further specified. Most investigations have documented that whenever the basic structure of pulp periphery is destroyed, hard tissue formation takes place as a part of the wound healing mechanism. However, clinical data showed that despite the presence of hard tissue barrier, treatment failed due to a secondary pulp infection. Experimental approaches showed that hard tissue which is formed as a part of the healing process results in formation of dentinal bridges with numerous tunnels and defects. Since pulp cells express a wide spectrum of mineralized matrices, it is crucial to distinguish the various types of hard tissue barriers formed in various pathological conditions at the pulp-dentin complex [16].

The cellular events taking place after direct pulp capping treatment have been studied by using calcium hydroxide-based materials. Initially the pulpal cells proliferate and elaborate a collagenous matrix in close proximity to a firm zone of destroyed pulp due to the trauma and the high alkalinity of the capping material. Mineral salts precipitating in that necrotic zone and the newly produced collagen form the first zone of calcified matrix around the treated pulp area. Morphologically this zone is called fibrodentin, and very often it has osteotypic appearance and does not include any tissue specific cytodifferentiative event [16,20]. If subsequent reactions lead to wound healing, the dentinogenic potential of pulpal cells can be expressed. Cells migrate toward the superficial calcified zone, attach and display the cytological characteristics of odontoblastic lineage forming a tubular mineralized matrix in a polar predentin-like pattern [7,20]. It seems that the primitive calcified matrix plays a role in differentiation of odontoblast-like cells, and this action calls to mind the role of the basement membrane during primary odontoblast differentiation in mediating epithelial-mesenchymal interactions [11]. It offers a mechanical support for immobilization of pulpal cells, though accumulation of endogenous extracellular matrix molecules (fibronectin and TGFβ1 among others) playing a role in the terminal odontoblast-like cell differentiation has been also suggested [14,21]. In any case, fibrodentin/osteodentin matrix formation seems to be an intermediate step during the onset of reparative dentinogenesis as a part of the wound healing process.

In conclusion, the clinical exploitation of dentinogenic potential of pulp cells represent the rational basis of evaluating biological reliability of PCM [8]. It is now a necessity to put the evaluation of PCM into a broader perspective. The therapeutic validity of a given pulp treatment strategy has to be associated with two main biological properties of the PCM:

- Biocompatibility, defined as the absence of toxic effects which can interfere with the overall outcome of the inflammatory process in the pulp, and
- Biological specificity, defined as the ability to signal odontoblast-like cell differentiation and reparative dentin formation instead the indirect stimulation of wound healing with hard tissue formation.

The aim of the present article is to perform a literature review on the therapeutic validity of various materials used in vital pulp therapy over the last 6 decades, as is indicated by their specific dentinogenic activity. Since the detailed pulp response to the capping materials, including both newly formed mineralized tissue and the associated formative cells characterization, requires evaluation at the light or transmission electron microscopic level, the relative histological studies were only reviewed.

## 2. Data Sources and Resources Selection

This critical review is based on a comprehensive literature search using the Medline/Pubmed data base covering the period from 1949 to early 2015. The database search was performed using the keywords “pulp capping histology”, “pulpotomy histology”, “reparative dentin” and “osteodentin and pulp cup or pulpotomy”. Eligible for inclusion in this study were scientific articles that were published in the English language, with no limitations implemented by country of origin. The relevant papers included the abstracts and full text of clinical trials (original articles) that met the eligibility criteria. Unpublished research and studies that were reported only in abstract form editorials, review articles, letters to the Editor, clinical guidelines, *in vitro* studies and case reports were not considered for inclusion.

Titles and abstracts were screened and then full texts of all potentially relevant publications were obtained and reviewed by two independent reviewers (X, Y). Full paper copies of peer-reviewed papers were acquired electronically and cross references were further screened to identify relevant studies. Both reviewers were blinded to authors, journal and results. Any disagreements on study inclusion and exclusion criteria were discussed and resolved either by consensus or by consulting a third reviewer.

Inclusion of a study was dependent on having sufficient data regarding the type of the capping material used and the unit of observation (human permanent tooth *in vivo* or animal permanent dentition; primary teeth were excluded). The searches were also confined to articles presenting either clinical/radiographic data and/or histologic/histomorphometric evaluations of the post-operatively deposited tissue related to the material tested. The post-operatively deposited matrix was named with various terms, which were categorized into three types as follows:

**Unspecified matrix**, where the type of newly formed mineralized matrix was characterized as hard tissue/matrix, mineralized tissue/matrix, calcified tissue/matrix or dentin bridge.
**Osteotypic matrix**, where the type of newly formed mineralized matrix was characterized as osteodentin, fibrodentin, osteotypic hard tissue/matrix, atubular dentin or reparative dentin without any indication of tubular structure, and
**Dentin-like matrix**, where the type of newly formed mineralized matrix was characterized by its tubular structure and was named as reparative dentin, tertiary dentin, new dentin, dentin-like or tubular mineralized tissue/matrix, with indication of presence of elongated formative cells, or odontoblast-like cells or new odontoblasts.

### Review

One hundred fifty two studies were included in the final evaluation. In Table 1 the studies are shown with their reference data, species, capping material(s) used and the type of mineralized tissue(s) formed according to the categorization mentioned above.

**Table 1:** The 152 studies which have been included in the present systematic review. Data concerning the used experimental model(s), capping material(s) and the type of newly formed mineralized tissue(s) are shown. Abbreviations: CH calcium hydroxide-based material, MTA mineral trioxide aggregates-based material, PC Portland cement-based material, BD biodentine, CP calcium phospate-based material, HA hydroxyapatite-based material, BG bioactive glass-based material, EMP enamel matrix protein-based material, FS ferric sulphate, FC formocresol, BMA bioactive molecule-based application, ZOE zinc oxide & eugenol-based material, RS glass ionomer and resin-based materials, U unspecified mineralized tissue, OSD osteotypic matrix, DL dentin-lik matrix.

References	Model	Capping Material(s)	Type of Matrix
Zhang *et al.* [22]	Rat	MTA, PC	DL
Cannon *et al.* [23]	Monkey	RS, PC	U
Swarup *et al.* [24]	Human	CP, MTA, CH	DL
Han *et al.* [25]	Rat	MTA	DL
Tziafa *et al.* [26]	Pig	MTA, BD	OSD + DL
Obeid *et al.* [27]	Dog	MTA, CP, Other	U
Nowicka *et al.* [28]	Human	BD, MTA	OSD + DL
Hutcheson *et al.* [29]	Human	MTA	U
Omar *et al.* [30]	Dog	FS, Other	U
Sushynski *et al.* [31]	Human	FS, MTA	DL
Nowicka *et al.* [32]	feline	RS, CH	DL
Cardoso-Silva *et al.* [33]	Human	MTA	U
Fransson *et al.* [34]	Human	BMA, CH	OSD + DL
Shahravan *et al.* [35]	Human	MTA	U
Zarrabi *et al.* [36]	Human	MTA, other	DL
Shayegan *et al.* [37]	Pig	HA, CH, FC	U
Zealand *et al.* [38]	Human	FC, MTA	U
Zarrabi *et al.* [39]	Human	MTA, other	DL
Parolia *et al.* [40]	Human	Other, MTA, CH	DL
Sakai *et al.* [41]	Human	MTA, PTC	U
Shayegan *et al.* [42]	Pig	CP, CH, MTA, PTC	U
Accorinte *et al.* [43]	Human	MTA	U
Kiatwateeratana *et al.* [44]	Human	EMP, CH	DL
Accorinte *et al.* [45]	Human	MTA, CH	U
Accorinte *et al.* [46]	Human	MTA, CH	U
Moretti *et al.* [47]	Human	MTA, CH, FC	U
Sawicki *et al.* [48]	Human	MTA, CH	U
Nair *et al.* [49]	Human	MTA or CH	U
Lu *et al.* [50]	Human	RS, CH	DL
Min *et al.* [51]	Human	MTA, CH	DL
Fernandes *et al.* [52]	Human	CH, RS	DL
Qudeimat *et al.* [53]	Human	MTA, CH	U
Tziafas *et al.* [54]	Dog	RS, CH, Other	U
Elias *et al.* [55]	Human	CH, RS	DL
Iwamoto *et al.* [56]	Human	MTA, CH	U
Caicedo *et al.* [57]	Human	MTA	U
Silva *et al.* [58]	Human	RS, CH	DL
Piva *et al.* [59]	Human	CH	OSD + DL
Olsson *et al.* [60]	Human	EMP, CH	U
Markovic *et al.* [61]	Human	FC, CH	U
Koliniotou & Tziafas. [62]	Dog	RS, CH	U
Maroto *et al.* [63]	Human	MTA	U
Suzuki *et al.* [64]	Rat	RS, Other	DL
Accorinte *et al.* [65]	Human	CH, RS	DL
Menezes *et al.* [66]	Dog	MTA, PTC	U
Agamy *et al.* [67]	Human	MTA, FC	DL
Iohara *et al.* [68]	Pig	BMA	DL
Nakashima *et al.* [69]	Dog	BMA	DL
Salako *et al.* [70]	Human	BG, MTA, FS, FC	DL
Hörsted-Bindslev *et al.* [71]	Human	RS, CH	DL
Scarano *et al.* [72]	Human	RS, CH, other	DL
Tziafas *et al.* [73]	Dog	MTA	OSD + DL
Tziafas *et al.* [74]	Dog	BMA, Other	OSD + DL
Kitasako *et al.* [75]	Monkey	RS, CH	DL
Murray *et al.* [76]	Monkey	CH, RS	DL
Hafez *et al.* [77]	Monkey	RS, CH	DL
Six *et al.* [78]	Rat	BMA, CH	OSD + DL
Nakamura *et al.* [79]	Pig	CH, EMP	OSD + DL
Tziafas *et al.* [80]	Dog	BMA, HA, CH, Other	OSD + DL
Goldberg *et al.* [81]	Rat	BMA, CH	OSD + DL
Lovschall *et al.* [82]	Rat	BMA, CH	DL
Rutherford. [83]	Rat	BMA	DL
Blanko *et al.* [84]	Human	CH	U
Pereira *et al.* [85]	Human	CH, RS	DL
Decup *et al.* [86]	Rat	BMA, CH	DL
Waterhouse *et al.* [87]	Human	FC, CH	DL
Hayashi *et al.* [88]	Rat	CP	OSD + DL
Kitasako *et al.* [89]	Monkey	RS	U
Hebling *et al.* [90]	Human	RS, CH	U
Tarim *et al.* [91]	Monkey	RS, ZOE, CH	U
Tziafas *et al.* [92]	Dog	BMA	OSD + DL
Tziafas & Papadimitriou. [93]	Dog	BMA	OSD + DL
Jepsen *et al.* [94]	Pig	BMA, CH	OSD + DL
Ford *et al.* [95]	Monkey	MTA, CH	U
Tziafas *et al.* [96]	Dog	CH	OSD + DL
Yoshiba *et al.* [97]	Human	CH	OSD + DL
Tziafas *et al.* [98]	Dog	BMA	OSD + DL
Sasaki & Kawamata-Kido. [99]	Rat	HA, CH	OSD + DL
Oguntebi *et al.* [100]	Pig	BG, CH, BMA, Other	U
Yoshimine *et al.* [101]	Rat	CP, CH	U
Yoshiba *et al.* [102]	Monkey	CP, CH, Other	U
Tziafas *et al.* [103]	Dog	BMA, CH	OSD + DL
Nakashima. [104]	Dog	BMA	OSD + DL
Tziafas *et al.* [105]	Dog	BMA	OSD + DL
Oguntebi *et al.* [106]	Pig	BG, BMA, CH	DL
Imai *et al.* [107]	Rat	CP, CH	OSD + DL
Lianjia *et al.* [108]	Bovine	BMA	OSD + DL
Rutherford *et al.* [109]	Monkey	BMA, CH	OSD + DL
Robson & Katz. [110]	Rat	BMA	OSD + DL
Inoue & Shimono. [111]	Rat	RS	U
Tziafas *et al.* [112]	Dog	BMA	OSD + DL
Tziafas *et al.* [113]	Dog	BMA	OSD + DL
Jaber *et al.* [114]	Rat	HA, CH	DL
Furusawa *et al.* [115]	Human	CP	U
Mjor *et al.* [116]	Monkey	CH	DL
Fitzgerald & Heys. [117]	Human	CH	U
van Mullem. [118]	Pig	Untreated	OSD + DL
Nakashima. [119]	Dog	BMA	OSD + DL
Smith *et al.* [120]	Ferret	BMA	OSD + DL
Fitzgerald *et al.* [121]	Monkey	CH	U
Ikami *et al.* [122]	Monkey	CP	U
Tziafas & Kolokuris. [123]	Dog	BMA	OSD + DL
Heys *et al.* [124]	Monkey	CH, Other	U
Nakashima. [125]	Dog	BMA	OSD + DL
Tziafas. [126]	Dog	CH, other	U
Oguntebi *et al.* [127]	Monkey	BMA, ZOE	U
Tziafas & Molyvdas. [128]	Dog	CH	OSD + DL
Jean *et al.* [129]	Pig	CP, HA, CH	OSD + DL
Cvek *et al.* [130]	Monkey	CH, other	OSD + DL
Cox *et al.* [131]	Monkey	RS, others	DL
Heide & Kerekes. [132]	Monkey	CH	U
Heide & Kerekes. [133]	Monkey	CH	U
Cox & Bergenholtz. [134]	Monkey	CH	OSD + DL
Schroder U. [20]	Human	CH	OSD + DL
Cox *et al.* [135]	Monkey	CH	U
Fuks *et al.* [136]	Monkey	BMA	OSD + DL
Goldberg *et al.* [137]	Human	CH, CP	U
Heide & Mjor. [138]	Human	Others	U
Garcia-Godoy *et al.* [139]	Dog	FC	U
Cox *et al.* [140]	Monkey	CH	U
Heys *et al.* [141]	Monkey	CH, ZOE, CP, Others	DL
Inoue *et al.* [142]	Rat	BMA	U
Horsted *et al.* [143]	Monkey	CH, Other	U
Dick & Carmichael. [144]	Dog	BMA	U
Fitzgerald. [145]	Monkey	CH	OSD + DL
McWalter *et al.* [146]	Monkey	CH, Others	U
Heller *et al.* [147]	Monkey	CP, CH	U
Cotton. [148]	Rat	CH, ZnOE	U
Cotton. [149]	Rat	CH	U
Tronstad. [150]	Monkey	CH	U
Schroder & Sundstrom. [151]	Human	CH	OSD + DL
Schroder. [152]	Human	CH	OSD + DL
Sella *et al.* [153]	Rat	CH	U
Mc Walter *et al.* [154]	Monkey	CH, Other	U
Stanley & Lundy. [155]	Human	CH	U
Anneroth & Bang. [156]	Monkey	BMA	U
Tronstad & Mjor. [157]	Monkey	CH, ZOE	U
Ulmansky *et al.* [158]	Human	CH	U
Schroder & Granath. [159]	Human	CH	OSD + DL
Berkman *et al.* [160]	Human	Others	U
Schroder & Granath. [161]	Human	CH	OSD + DL
Ulmansky *et al.* [162]	Human	CH, other	DL
Langer *et al.* [163]	Human	CH, ZOE	U
Bhaskar *et al.* [164]	Rat	CH, other	U
Kakehashi *et al.* [165]	Rat	Other	DL
Kakehashi *et al.* [18]	Rats	Untreated	U
Pisanti & Sciaky. [166]	Dog	CH	U
Sciaky & Pisanti. [167]	Dog	CH	U
Kalnins & Frisbie. [168]	Human	Untreated	U
Berman & Massler. [169]	Rat	CH, ZOE	U
Nyborg. [170]	Human, Dog	CH	U
Glass & Zander. [18]	Human	CH, ZOE, other	U

## 3. Data Analysis

Experimental model: Sixty studies (39.2%) were performed on human teeth, while the following species were also used for the animal experimentation: non-human primates in 31 studies (20.2%), non-primates in 39 studies (25.4%), including dogs, pigs, felines, and bovine species, and rodents in 25 studies (16.3%), including rats, mice and ferrets.

Capping material: Ninety-three studies (60.7%) used calcium-hydroxide-based materials as testing or control material. After introduction of MTA as a capping material in 1996, 35 studies (22.8%) used tricalcium-silicate-based materials. It is noteworthy that of the 54 studies published after 2003, MTA was used in 31 studies (57.4%). In 82 studies, other dental materials have been used: in 20 resin-based materials (13%), in 18 calcium phosphate-based materials (11.7%), in 8 zinc oxide eugenol-based materials (5.2%) and in 39 studies (25.5%) other materials (ferric, sulfate, formaldeyde, amalgam, *etc.*) were applied. In thirty-seven studies (24.2%), bioactive-molecule-based applications including growth factors, bone morphogenetic proteins, enamel proteins, *etc.*, were used.

Regarding the form and structure of the mineralized bridge, the type of hard tissue bridge was characterized as follows:

- In 74 (48.3%) studies, only the presence of a mineralized bridge was reported. The type of mineralized matrix was not adequately described and characterized as hard, mineralized or calcified tissue or dentin bridge.
- In 33 (21.6%) studies, the form of the mineralized bridge was evaluated according to morphological or molecular characterization of the newly formed matrix. The type of mineralized matrix was categorized as a dentin-like matrix, but without any information on whether this type of mineralized matrix characterized the whole structure of the mineralized bridge.
- In 46 (30.1%) studies, the form of the mineralized bridge was evaluated according to morphological or molecular characterization of the newly formed matrix, and two types of mineralized matrix have been reported. A firm zone of a mineralized matrix, which was categorized as an osteotypic mineralized matrix, was always followed by the mineralized matrix categorized as dentin-like matrix.

**Figure 1:**
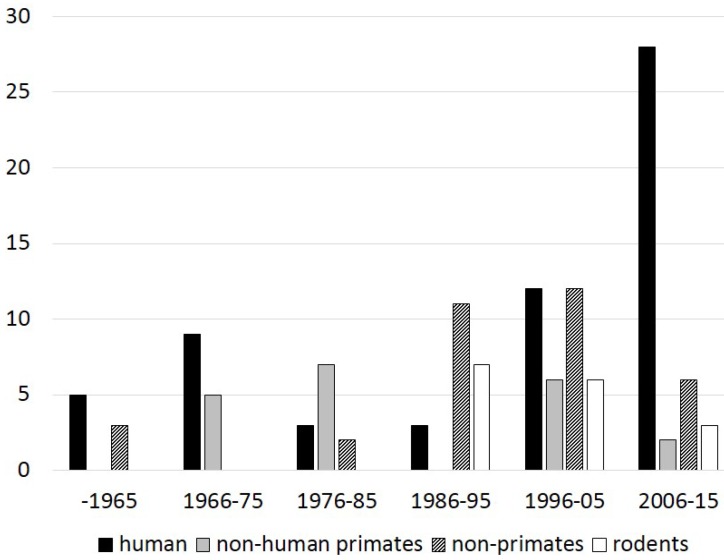
Number of experimental pulp capping histological studies (y-axis) performed in different experimental models per decade.

**Figure 2:**
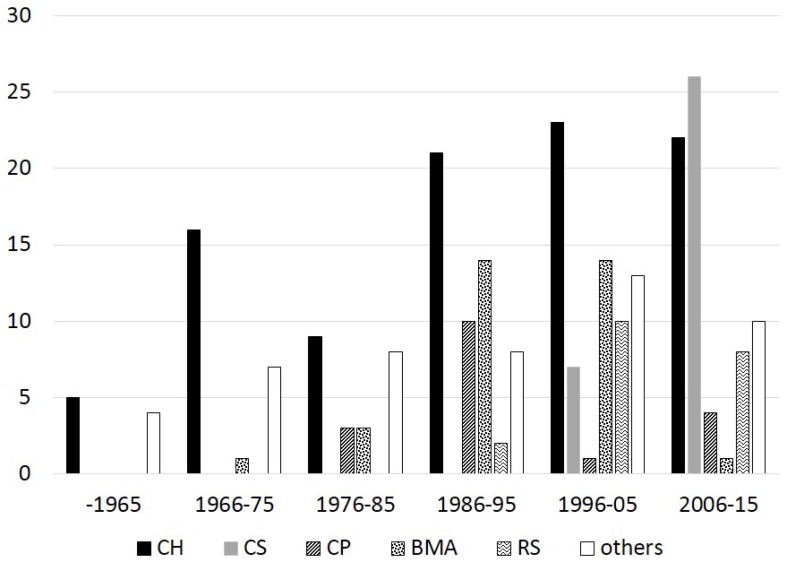
Number of experimental pulp capping histological studies (y-axis) performed by using different capping materials per decade. CH calcium hydroxide-based materials, CS calcium silicate-based materials, CP calcium phosphate-based materials, BMA bioactive molecule-based applications, RS resin-based materials.

**Figure 3:**
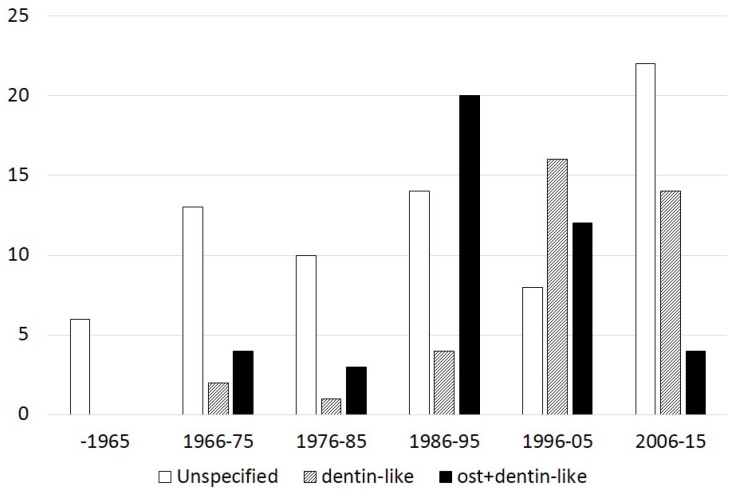
Number of experimental pulp capping histological studies (y-axis) with different types of post-operatively formed hard tissue, as has been previously described, per decade.

The data per decade concerning the experimental model, capping materials used and type of hard tissue bridge are shown in Figure 1, Figure 2 and Figure 3, respectively.

## 4. Conclusions

It is well-accepted that both the presence and (especially) the quality of hard tissue barrier are important prognostic factors for the success of a pulp capping treatment [8]. Thus, the ultimate goal of the direct pulp capping procedure and the optimal end result of an ideal PCM in the exposed pulp might be the complete reconstitution of the anatomy of pulp periphery. Since primary dentin cannot be formed post-developmentally, the differentiation of odontoblast-like cells forming reparative dentin in a predentin-like pattern is an essential biological pre-requirement for the complete restoration of dentin-pulp complex integrity, the only predictable way to achieve the successful outcome of vital pulp therapy. Differentiation of odontoblast-like cells takes place as a part of the wound healing mechanism, or as an effect of specific signaling molecules on pulp cells. Traditional pulp capping materials stimulate or enhance the wound healing mechanism, forming a heterogeneous hard tissue bridge composed of osteodentin-fibrodentin and reparative dentin. Several experimental attempts showed that the application of exogenous signaling molecules offers opportunities for the development of new therapies. Furthermore, the presence of a cocktail of growth factors in the dentin extracellular matrix provides a tool for an endogenous signaling mechanism to modulate cellular events taking place during tertiary dentinogenesis. The ability of biomaterials to release bioactive molecules from the dentin has been documented by robust studies, providing a basis for new significant tasks in developing novel therapeutic approaches in the near future.

Addressing delivery matters need to be considered before their introduction in clinical practice. In the shorter term, the group of traditional PCMs remains as the only therapeutic possibility for effective hard tissue bridge formation at the exposure site. The ability of traditionally used PCMs to stimulate the hard tissue bridge has been documented in experimental histological pulp capping studies by using different assessment criteria. It must be assumed that the absence of uniform criteria is the reason for the fact that our knowledge on the specificity of PCMs to induce dentinogenic events is still very limited. Thus, further data are required to fully understand how the wound healing mechanism after application of a PCM could be designed as a time- and space-limited process to provide the optimal end result, avoiding incomplete restoration of pulp periphery or uncontrolled pulp obliteration.

Data from the present systematic review have shown that only 30.2% of the 152 experimental histological pulp capping studies described the heterogenic nature of the hard tissue bridge formation, while the 48.2% and 21.6% of the studies reported an homogenous hard tissue bridge characterized as hard, mineralized or calcified tissue and dentin bridge or dentin-like matrix, but without any additional information on its structural characteristics, respectively. It seems to be quite disappointing that there is a declining percentage of studies using specific criteria to characterize the hard tissue bridge over the last 30 years. As can be seen in Figure 3, 52.6%, 32.4% and 10.2% of the studies used specific characterization of the type of post-operatively formed hard tissue in the periods 1986–1995, 1996–2005 and 2006–2015, respectively. In the present study, our knowledge about the nature of pulp wound healing mechanisms is highlighted as a critical requirement in the selection of the appropriate vital pulp therapy procedure and capping materials in various clinical conditions. Additionally, it is suggested that an international consensus must be reached on selected mandatory criteria to optimally characterize the reparative events in vital pulp therapy. 
